# The association between perceived COVID-19-related discrimination and probable depression among pregnant women in the post-pandemic era: a cross-sectional study

**DOI:** 10.3389/fpubh.2025.1588589

**Published:** 2025-06-11

**Authors:** Peiqin Liang, Yu Chen, Runjun Li, Yan Liu, Ribo Xiong

**Affiliations:** ^1^Department of Gynecology & Obstetrics, The Third Affiliated Hospital of Southern Medical University, Guangzhou, China; ^2^School of Nursing, Southern Medical University, Guangzhou, China; ^3^Department of Rehabilitation, The Seventh Affiliated Hospital, Southern Medical University, Foshan, China

**Keywords:** coronavirus, antenatal depression, epidemic, risk factors, China

## Abstract

The 2019 coronavirus disease (COVID-19) has substantially influenced the physical and psychological wellbeing of populations who survived the pandemic. Discrimination against infected individuals has been a public health concern and has been identified as a risk factor for mental health issues. However, limited empirical evidence is available to support its role in psychological disorders during the prenatal period. The current study aims to investigate the association between COVID-19-related discrimination and the likelihood of experiencing probable depression among women in their early pregnancy. A cross-sectional study was conducted from 1 May 2023 to 31 August 2023 using an anonymous online questionnaire distributed to women in their first trimester of pregnancy in the antenatal care clinic of three hospitals. The Edinburgh Postnatal Depression Scale was used to screen antenatal depression. The logistic regression model was used to assess the relationship between COVID-19-related discrimination and antenatal depression. A total of 3,608 pregnant women were enrolled in this study, of whom 657 screened positive for antenatal depression (AND), resulting in a prevalence of 18.2%. More than one-third of the participants experienced COVID-19-related discrimination. The logistic regression analysis demonstrated a greater prevalence of probable depression among pregnant women who experienced COVID-19-related discrimination compared with those who did not. This study provided novel evidence for the association between COVID-19-related discrimination and probable depression in pregnant women, highlighting an associated factor for supportive interventions that may be relevant in the post-pandemic era.

## Introduction

The 2019 coronavirus disease (COVID-19) is a global pandemic with marked physical and mental health consequences (1). While most patients recovered following acute infection, evidence indicated that, in a large population of survivors, impairments in the function of one or more organs may persist, a phenomenon referred to as long COVID-19 (2). Published reports indicate that approximately 10–20% of patients experienced lingering symptoms for weeks to months, posing a significant public health concern (3). Long-lasting sequelae of COVID-19 survivors include fatigue, myalgia, dyspnea, sleep disturbance, cognitive deficits, anxiety, depression, and post-traumatic stress disorder (PTSD) (2). A cohort study with a 6-month follow-up duration conducted in Wuhan, China demonstrated that 23% of patients continued to experience anxiety or depression (4). Another cross-sectional study of COVID-19 survivors revealed that the prevalence rates of anxiety, depression, and PTSD were 27.1, 36.2, and 15.2%, respectively, 1 year after infection (5). Despite psychiatric sequelae among COVID-19 survivors not being the most common symptoms in the >12-month interval, as suggested by a meta-analysis on the prevalence of long COVID-19 symptoms at different follow-up periods (6), reports have indicated that the mental health status of COVID-19 survivors continued to worsen than those who had never been infected (2, 6). Moreover, results from longitudinal studies demonstrated that these neuropsychiatric symptoms were particularly frequent in female survivors after a longer interval (7). The underlying mechanisms of the psychiatric consequences of COVID-19 are likely multifactorial and might include virus-driven alterations, dysregulated immune reaction, intensive care, quarantine, and stigma (8). Therefore, it is important to highlight these psychological sequelae in female COVID-19 survivors and identify their influencing factors.

People who have survived COVID-19 have been subjected to an increase in discrimination worldwide (9). Discrimination is a social process set up to exclude those who are suspected to have or diagnosed with COVID-19 and may pose a threat to effective social living in society (9). A nationwide cross-sectional study conducted in China during the early stages of the pandemic demonstrated that the COVID-19 outbreak led to region-based and disease-related discrimination against individuals both at the individual and community levels (10). As COVID-19 spread globally, anti-Asian discrimination became widespread, particularly in Western countries, due to the presumed origin of the virus (11). Discrimination also posed a serious threat to COVID-19 survivors and was shown to be associated with mental health problems such as psychological distress, anxiety, depression, sleeping difficulty, and PTSD symptoms (9).

For pregnant women specifically, antenatal depression (AND) is one of the most common mental health problems during pregnancy, with a prevalence ranging from 11.9 to 19.7% (12). AND is characterized by persistent low mood, feelings of guilt or worthlessness, lack of enthusiasm, and feeling isolated or isolated (12). Evidence has shown that AND has adverse effects on obstetric and neonatal outcomes, such as premature delivery, cesarean section, low birth weight, and postpartum depression (13). Studies also suggest the transmission of psychopathology from a mother to her offspring (14). Therefore, the prenatal period is a good opportunity for health professionals to prevent and control.

Recent evidence has highlighted the critical role played by discrimination in the onset and maintenance of depression. Nevertheless, limited information is available on how COVID-19-related discrimination affects depressive symptoms in pregnant women. This finding is particularly pertinent, given that this period is characterized by unique episodes, such as hormonal alteration, social role transformation, and insomnia, which could potentially interact with perceived discrimination. Therefore, this study aimed to examine the association between COVID-19-related discrimination and probable AND among pregnant women.

## Methods

### Study design and setting

This study was carried out in Guangzhou, China. Guangzhou is the third largest metropolis in China with a population of more than 16 million. The city has witnessed the most complex and severe COVID-19 prevention and control situation during the period from 2020 to 2022. On 9 December 2022, the Chinese government announced that the epidemic ended.

A cross-sectional study was conducted from 1 May 2023 to 31 August 2023 at three public hospitals. These three facilities have the largest antenatal care clinics in Guangzhou, and together, they serve more than 12,000 pregnant women annually before the outbreak of COVID-19.

### Participants

Women in their first trimester who were registered at any of the facilities were offered the opportunity to participate in the study. The inclusion criteria were as follows: (1) Chinese nationality and (2) willingness to participate. Participants who met any of the following exclusion criteria were excluded: (1) a history of clinically diagnosed psychiatric disorder before 1 January 2020, namely, the outbreak of COVID-19 in China by checking medical records; (2) a history of clinically diagnosed psychiatric illnesses due to COVID-19 by checking medical records; and (3) difficulty cooperating with the questionnaire survey.

### Sample size

The sample size was determined using the single population proportion based on a 5% level of significance with a 5% margin of error, assuming a prevalence of antenatal depression of 20.5% (15). A minimum sample size of 1,023 individuals is required with 80% power at the 5% significance level, taking into account 5% non-response. This study included 3,608 women, providing a power of 0.92 to detect significant differences, as calculated by Gpower software. The number of participants from each hospital was determined based on the average of the previous 4 months’ antenatal care clinic flow of each institution. The sample size was proportionately allocated as 1,210, 1,245, and 1,153, respectively.

### Data collection

During the period from 1 May 2023 to 31 August 2023, women who attended antenatal care were invited by nurses using WeChat, a popular messaging app in China. Digital informed consent was obtained from all individuals to ensure their voluntary participation. We distributed self-reported anonymous questionnaires to the participants through an online survey platform (“SurveyStar”, Changsha Ranxing Science and Technology, Shanghai, China). Once the questionnaires were completed and submitted, research assistants collected the data using the computer version of “SurveyStar” on the desktop computer or laptop. The research assistants were available throughout the survey to assist participants, and regular supervision and feedback were provided daily by the principal researcher during the data collection period.

### Variable measurement

#### Explanatory variable

COVID-19-related discrimination was evaluated by asking participants two questions. (1) “Have you or your family ever experienced verbal discrimination related to COVID-19?” and (2) “Have you ever perceived discrimination related to COVID-19?”. Participants responded with a “yes” or a “no” response. Endorsing either of these experiences was classified as COVID-19-related discrimination. We developed these two items specifically for this survey, drawing on Berger’s HIV Stigma Scale for the dimensions of personalized stigma (patients experience and feelings when they realized that they were infected or suspected of being infected with coronavirus and had to be isolated/quarantined) and concern with public attitudes (their opinion of how they were and are being treated by everyone) (9, 16). This two-item scale was previously used in assessing COVID-19-related discrimination among healthcare workers (9, 16). When applied to pregnant women, a group discussion was conducted with 30 pregnant women at three hospitals, and psychiatrists who treated perinatal mental health issues carefully reviewed the items. The investigators also reviewed the items. The investigators and pregnant women involved in the development process of this scale did not participate in the survey.

#### Outcome variable

We assessed AND using the Edinburgh Postnatal Depression Scale (EPDS). The 10-item instrument is rated on a 3-point Likert scale from 0 to 3, with a cutoff point of 13. Although originally developed for postpartum depression, the EPDS has also been validated for AND screening (17). The Chinese version had satisfactory reliability and validity, with a Cronbach’s *α* of 0.96 and a correlation coefficient of 0.79 (18).

### Covariates

Two sets of covariates were included in the analysis to control for potential confounders: (1) Sociodemographic factors: age (continuous), education (tripartite, college or senior high school or junior high school), employment status (dichotomous, employment or unemployment), engaged in COVID-19-related work (dichotomous, yes or no), marital status (dichotomous, married or unmarried), average monthly household income (tripartite, CNY¥10,000–19,000 or CNY¥20,000–29,000 or CNY¥ ≥ 30,000), living arrangement (dichotomous, living with someone or living alone), perceived family socioeconomic status (dichotomous, stable or unstable), and marital relationship (tripartite, poor or moderate or satisfying). (2) Clinical information: COVID-19-related hospitalization (dichotomous, yes or no), gestational age (continuous), parity (tripartite, 0 or 1 or ≥2), history of abortion (dichotomous, yes or no), planned pregnancy (dichotomous, yes or no), and pregnancy-related complications (dichotomous, yes or no).

### Statistical analysis

Statistical analysis was performed in the SPSS 20.0 statistical package. A *p*-value of less than 0.05 was considered statistically significant (two-tailed tests). The characteristics of participants with and without probable AND were compared using a chi-squared test (for categorical variables) or *t*-test (for continuous variables or the Wilcoxon rank-sum test if the distribution was skewed). Collinearity between variables was evaluated, and no significant tolerance issues were observed. The logistic regression model was used to assess how COVID-19-related discrimination experience influenced the likelihood of probable AND, controlling for covariates.

### Ethical considerations

Ethics approval was obtained from the Research Ethics Board of The Seventh Affiliated Hospital, Southern Medical University (No. 1002507). Before participating in the study, participants who provided consent to the study were required to fill an online survey and click the submit button at the end. Responses to the questionnaire were recorded and analyzed anonymously.

## Results

Among 3,608 pregnant women enrolled in the assessment, 657 screened positive for AND with EPDS at a cutoff point of 13, thus resulting in a prevalence of 18.2%. In addition, 54.0% of the participants were aged between 25 and 34 years. A majority of the participants (69.3%) had completed senior high school or more. Moreover, 88.0% of the participants were married. Regarding employment, 58.0% of the participants were employed. The largest proportion (43.1%) of households earned between CNY¥10,000 and 19,000 per month, followed by those who earned between CNY¥20,000 and 29,000 (42.4%). Nearly one-third of the respondents reported COVID-19-related discrimination.

The results of the chi-squared or Student’s *t*-test are shown in Table 1. Women with probable AND were more likely to live alone, have unstable family socioeconomic status, and endorse COVID-19-related discrimination experiences than those without probable AND.

**Table 1:** Characteristics of women with antenatal depression.

Factors	EPDS<13 (2951)		EPDS≥13 (657)		*χ*^2^/*t*	*p*
	*n*	%	*n*	%		
Age					0.032	0.984
≤24	732	24.8	168	25.5		
25 ~ 34	1,594	54.0	353	53.8		
≥35	625	21.2	136	20.7		
Educational level					0.075	0.963
Junior high school or less	776	26.3	177	26.9		
Senior high school	1,263	42.8	285	43.4		
College or more	912	30.9	195	29.7		
Marital status					0.131	0.754
Married	2,591	87.8	585	89.0		
Unmarried	360	12.2	72	11.0		
Employment					0.020	0.918
Yes	1709	57.9	385	58.6		
No	1,242	42.1	272	41.4		
Engaged in COVID-19-related work					0.269	0.793
Yes	1,092	37.0	242	36.8		
No	1859	63.0	415	63.2		
Average monthly household income					0.105	0.949
CNY10,000 ~ 19,000	1,275	43.2	281	42.8		
CNY20,000 ~ 29,000	1,243	42.1	285	43.4		
≥ CNY30,000	433	14.7	91	13.8		
Living arrangement					4.341	0.040
Living with someone	1,540	52.2	413	62.8		
Living alone	1,411	47.8	244	37.2		
COVID-19-related discrimination					5.134	0.025
Yes	936	31.7	263	40.0		
No	2015	68.3	394	60.0		
Perceived family socioeconomic status					10.625	0.002
Stable	1986	67.3	335	51.0		
Unstable	965	32.7	322	49.0		
Marital relationship					0.017	0.991
Poor	319	10.8	72	11.0		
Moderate	1,443	48.9	317	48.3		
Satisfying	1,189	40.3	268	40.7		
COVID-19-related hospitalization					0.003	1.000
Yes	159	5.40	36	5.5		
No	2,792	94.6	621	94.5		
Gestational age	11.3 ± 0.9		10.9 ± 1.6		12.045	2.361
Parity					0.007	0.996
0	965	32.7	217	33.1		
1	1,594	54.0	353	53.8		
≥2	392	13.3	87	13.1	≥2	
History of induced abortion					0.007	1.000
Yes	944	32.0	213	32.4		
No	2007	68.0	444	67.6		
Planned pregnancy					0.002	1.000
Yes	1880	63.7	417	63.4		
No	1,071	36.3	240	36.6		
Pregnancy-related complications					0.003	1.000
Yes	159	5.40	36	5.5		
No	2,792	94.6	621	94.5		

CNY, Chinese Yuan.

The results of the logistic regression model that examined the relationships between COVID-19-related discrimination and probable AND are shown in Table 2. The model controlled for sociodemographic factors and clinical information. COVID-19-related discrimination experiences were significantly associated with probable AND (OR = 2.017, 95%CI: 1.642 ~ 3.135).

**Table 2:** Analysis based on the logistic regression model.

Predisposing factors	*B*	*S E*	*p*	*OR*	*95% CI*
Living arrangement					
Living alone	0.316	0.178	0.093	1.549	0.725 ~ 1.912
Perceived family socioeconomic status					
Unstable	0.175	0.009	0.126	1.284	0.493 ~ 1.686
COVID-19-related discrimination	0.359	0.081	0.000	2.017	1.642 ~ 3.135

## Discussion

Although COVID-19-related discrimination can contribute to the development of psychiatric symptoms, little is known about its role during the antenatal period. Findings from cross-sectional studies or cohort studies have primarily focused on post-traumatic stress disorder (PTSD) among COVID-19 survivors or healthcare workers (4, 9). For women, the transition to expectant motherhood is a challenging period and has been considered a window of increased vulnerability for mental illness (15). This is one of the first studies examining the association between COVID-19-related discrimination and probable AND among pregnant women in the post-pandemic era.

As hypothesized, COVID-19-related discrimination was associated with subsequent probable AND in pregnant women. This finding extends the literature on adverse outcomes of perceived COVID-19-related discrimination, adding more specific evidence relevant to the mental health of women who have recently been pregnant. This finding is consistent with previous studies, which demonstrated that stigma was a risk factor for psychological problems among COVID-19 survivors (4, 9). In recent years, perceived discrimination as one type of stressor has received growing attention. For pregnant women specifically, several cross-sectional studies revealed associations between depressive symptoms and different types of perceived discrimination, including racial discrimination among Black pregnant women (19), gender and economic discrimination among African-American (20), and lifetime discrimination among low-income, inner-city women in Philadelphia (21). A variety of bio-psycho-social models have been proposed to underlie this relationship. On the one hand, from the perspective of traumatic stress theory, COVID-19-related discrimination experience may be traumatic if one perceives it as a threat to their physical or emotional wellbeing and if one is out of their control (22). In such a case, these unpleasant experiences may increase their negative perceptions (i.e., a potential source of disease and a threat to effective social activities and normal lives) about their intrinsic worth. This effect may be particularly likely for women in their early pregnancy, given that first-trimester pregnancy is accompanied by tremendous change and adjustment (i.e., physical discomfort and transformation in social roles) (23). Therefore, pregnant women experiencing such events may suffer from psychological distress when reflecting on these circumstances, leading to depressive symptoms. Moreover, drastic hormonal changes in early pregnancy do have an impact on increased emotional reactivity (21). Over-reactivity to COVID-19-related discrimination may eventually lead to dysfunction of bodily systems, including those controlling mental health. On the other hand, at community levels, negative perceptions regarding those who had experienced COVID-19-related stigma could have a harmful effect on mental health (14). A previous study indicated that discrimination against individuals could lead to a feeling of guilt about contracting the virus, which could discourage them from utilizing prenatal health services, postponing medical tests, or preventive screenings, worsening their depressive symptoms (24). In addition, this discrimination was also associated with social isolation, which might dissuade them from openly sharing their thoughts and emotions, potentially intensifying their depressive symptoms (24). Thus, we hypothesized that pregnant women who experienced COVID-19-related discrimination might experience high levels of psychological stress, which, in turn, would predict probable AND.

Our findings linking COVID-19-related discrimination to probable AND underscore the need for efforts aimed at reducing discrimination in the post-pandemic era. Several interventions have been suggested. For instance, mindfulness interventions aim to foster attention and awareness of present-moment experience. Cognitive-behavioral therapy helps individuals identify their stigma and engage in active and effective problem-solving. Moreover, given that COVID-19-related discrimination is a social process ingrained at both the individual and community levels, coordinated efforts to reduce the stigma of pregnant women are critical. A community rehabilitation program should be developed to facilitate positive interactions between the general and stigmatized populations and help them to reintegrate into society.

The present study has several limitations. First, a cross-sectional design precludes inferring a causal or directional association between COVID-19-related discrimination and probable AND. Second, we employed an instrument that has not been extensively validated to measure stigma among pregnant women, which may influence the estimates and generalizability. Third, there is potential bias stemming from unmeasured confounders, such as resilience to traumatic events, disease severity during COVID-19 infection, length of hospital stay, and vaccination history. Fourth, an online survey was conducted instead of the face-to-face interview, which may introduce sampling bias and limit the generalizability of the findings.

## Conclusion

COVID-19-related discrimination was associated with a higher prevalence of probable AND among pregnant women. Although further studies are necessary to confirm the impact of COVID-19-related discrimination on probable AND, this study indicated that perceived discrimination may be an important psychosocial correlate of probable AND among pregnant women in the post-pandemic era.

## Data Availability

The raw data supporting the conclusions of this article will be made available by the authors, without undue reservation.
