# Supplementary material for: The association between perceived COVID-19-related discrimination and probable depression among pregnant women in the post-pandemic era: a cross-sectional study
Source: Front Public Health. 2025 Jun 11;13:1588589. doi: 10.3389/fpubh.2025.1588589 (PMC12187655; doi:10.3389/fpubh.2025.1588589)
Supplement: Supplementary file 1 [file Table_1.docx]

|  | Unstandardized coefficient | |  | Standardized coefficient | *t* | *P* | *VIF* |
| --- | --- | --- | --- | --- | --- | --- | --- |
|  | β standard error | |  |  |  |  |  |
| Constant | -6.013 | 31.287 |  | - | -0.374 | 0.759 | - |
| Educational level | 1.138 | 0.130 |  | 0.625 | 13.121 | 0.057 | 1.793 |
| Employment status | 1.752 | 1.234 |  | 0.420 | 1.847 | 0.081 | 1.706 |
| Average monthly household income | 2.136 | 0.371 |  | 1.104 | 1.559 | 0.032 | 2.185 |
| Family socio-economic status | 1.978 | 1.223 |  | 0.318 | 1.107 | 0.064 | 2.450 |
| COVID-19-related hospitalization | 0.471 | 4.583 |  | 0.026 | 0.218 | 0.007 | 3.019 |
| COVID-19-related discrimination | -1.128 | 16.896 |  | -0.005 | -0.065 | 0.812 | 3.623 |

Appendix 1. Collinearity evaluation results between variables
